# Role of chronic *E. coli* infection in the process of bladder cancer- an experimental study

**DOI:** 10.1186/1750-9378-7-19

**Published:** 2012-08-08

**Authors:** Hala El-Mosalamy, Tarek M Salman, Abeer M Ashmawey, Nada Osama

**Affiliations:** 1 Biochemistry Department, Faculty of Pharmacy, Ain Shams University, Cairo, Egypt; 2 Biochemistry Department, Faculty of Pharmacy, Al-Azhar University, Cairo, Egypt; 3 Cancer Biology Department, National Cancer Institute, Cairo University, Cairo, Egypt; 4 Biochemistry Department, Faculty of Pharmacy, Modern Sciences and Arts University, Cairo, Egypt

**Keywords:** Bladder carcinogenesis, *E. coli*, NF-κBp65, Bcl-2, IL-6

## Abstract

**Background:**

Bladder cancer is a common malignancy in Egypt. A history of urinary tract infection can be considered as a risk factor for bladder cancer. *Escherichia coli (E. coli)* infection is responsible for 70% of urinary tract infection. This study aimed to evaluate the role of chronic *E. coli* infection during bladder carcinogenesis. In order to achieve this aim, we investigated the histopathological changes in bladder tissue and measured the level of nuclear factor kappa p65 (NF-κBp65), Bcl-2 and interleukin 6 (IL-6) in four groups each consisting of 25 male albino rats except of control group consisting of 20 rats. The first group was normal control group, the second group was infected with *E. coli*, the third group was administered nitrosamine precursor, and the forth group was infected with *E. coli* and administered nitrosamine precursor.

**Results:**

The histopathological examination revealed that *E. coli* infected group was able alone to produce some histopathological changes in bladder tissue and that nitrosamine precursor plus *E. coli* group showed highest incidences of urinary bladder lesions than the nitrosamine precursor group. NF-κBp65, Bcl-2 and IL-6 levels were significantly higher in nitrosamine precursor plus *E. coli* group than the other groups.

**Conclusion:**

These findings suggested that urinary bladder infection by *E. coli* may play a major additive and synergistic role during bladder carcinogenesis.

## Background

Bladder cancer is a common malignancy, worldwide; it is the seventh most prevalent cancer, accounting for 3.2% of all malignancies [1]. Carcinoma of the bladder is the most prevalent cancer in Egypt. At the national cancer institute, Cairo University, it constitutes 30.3% of all cancers [2,3]. Nitrate contamination of drinking water was reported as a risk of bladder cancer. Nitrates are endogenously reduced to nitrites, which through subsequent nitrosation give rise to highly carcinogenic N-nitroso compounds [4]. Other etiological factors implicated in the development and progression of bladder cancer includes urinary tract infections (UTIs) including bacterial, parasitic, fungal, and viral infections; urinary lithiasis and pelvic radiation [5]. Bacteria are the primary cause of UTIs, with the vast majority (70–80%) attributed specifically to infection with *E. coli*. A recurring theme in the link between bacterial infection and carcinogenesis is that of chronic inflammation, which is often a common feature of persistent infection [6,7]*.* One of the key molecules that link chronic inflammation and cancer is represented by the NF-κB family of transcription factors [7]*.* NF-κB activation induces the expression of more than 200 genes which have been shown to suppress apoptosis and induce cellular transformation, proliferation, invasion, metastasis, chemo-resistance, radio-resistance, and/or inflammation [8]. Altered expression of genes involved in suppression of apoptosis (i.e. Bcl-2 family members and inhibitor of apoptosis proteins), a key feature of cancer cells, is often due to deregulated NF-κB activity. The expressions of numerous cytokines that are growth factors for tumor cells such as interleukin 1β (IL-1β); tumor necrosis factor (TNF); epidermal growth factor (EGF) and IL-6 are also regulated by NF-κB [9]. IL-6 is a major proinflammatory cytokine that participates in inflammation-associated carcinogenesis [10]. Elevated plasma and urine levels of IL-6 have been demonstrated in cancer and inflammatory diseases of the urinary tract [11]. This study aimed to evaluate the possible role of *E. coli* infection during bladder carcinogenesis and the changes in NF-κB pathway and its related products.

## Results

All experimental protocols and procedures were approved by the Animal Ethics Committee of Cairo National Cancer Institute.

### Histopathological examination

**Figure 1:**
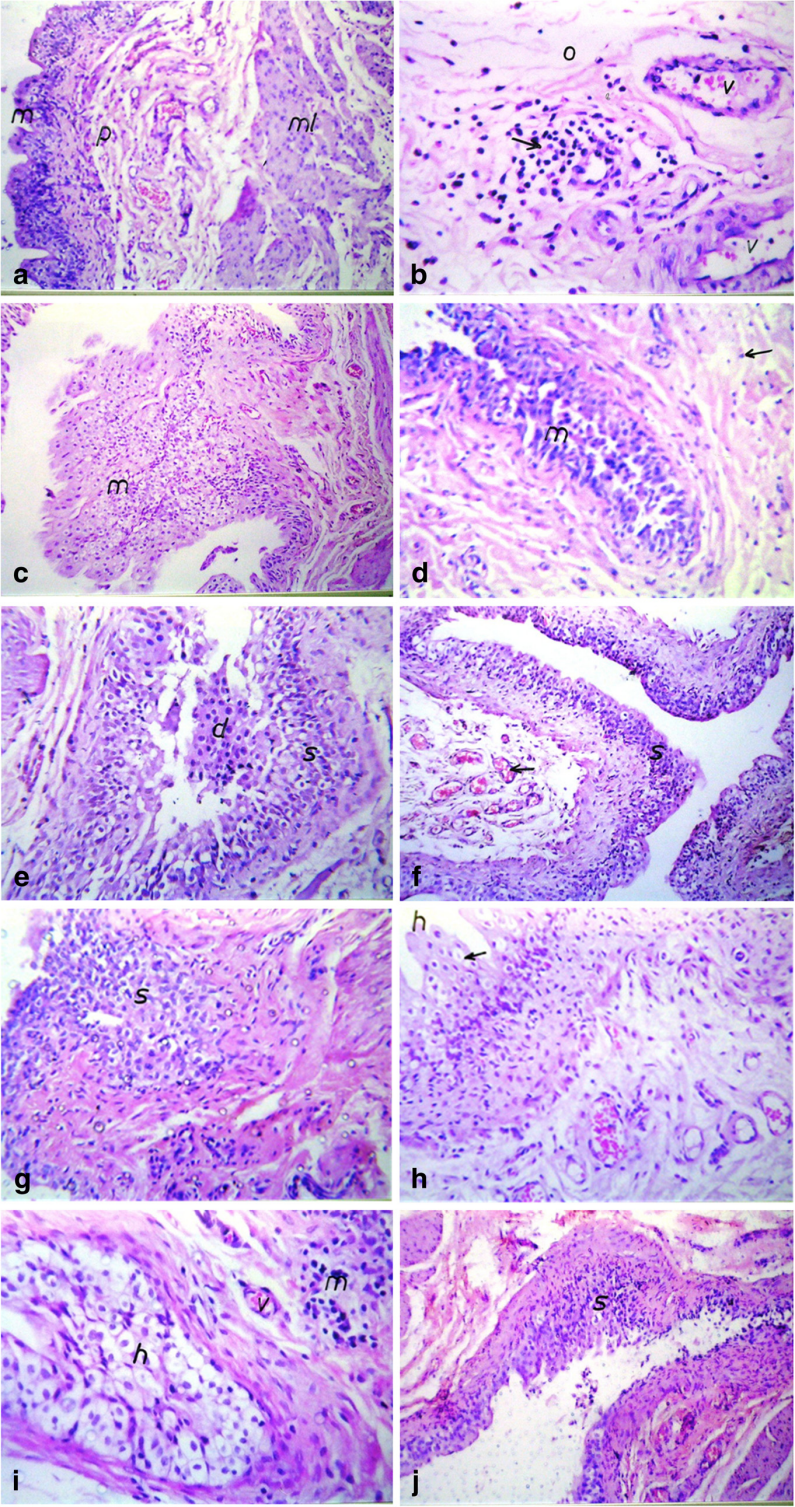
**Histopathological changes in different studied groups; control group (group I),*E. coli*group (group II), nitrosamine precursors group (group III), and*E. coli*and nitrosamine precursors group (IV). (a)**: Urinary bladder of rat in control group (group I) showing normal histological structure of the lining mucosal epithelium (m), underlining lamina propria (p) and muscularis (ml). **(b)**: Urinary bladder of rat in *E. coli* group (group II) at 3 months showing focal inflammatory cell infiltration (arrow) with sever dilatation of blood vessels in the lamina propria **(c)**: Urinary bladder of rat in *E. coli* group (group II) at 6 months showing focal hyperplasia and stratification in the mucosal epithelium (m). **(d)**: Urinary bladder of rat in *E. coli* group (group II) at 9 months showing dysplasia in the mucosal lining epithelium (m) with inflammatory cells infiltration in lamina propia (arrow). **(e)**: Urinary bladder of rat in nitrosamine precursors group (group III) at 3 months showing dysplasia in the mucosal lining epithelium **(d)** with degenerative changes (s). **(f)**: Urinary bladder of rat in nitrosamine precursors group (group III) at 6 months showing dysplasia in the mucosal epithelium (s) with congestion of newly formed blood capillaries in underlining lamina propria (arrow). **(g)**: Urinary bladder of rat in nitrosamine precursors group (group III) at 9 months showing dysplasia in the lining mucosal epithelium (s). **(h)**: Urinary bladder of rat in *E. coli* and nitrosamine precursors group (group IV) at 3 months showing hyperplasia with polyp formation (h) and degeneration (arrow) in mucosal lining epithelium. **(i)**: Urinary bladder of rat in *E. coli* and nitrosamine precursors group (group IV) at 6 months showing degeneration and dysplasia **(h)** in mucosal lining epithelium with inflammatory cells infiltration (m) in underlying lamina propria. **(j)**: Urinary bladder of rat in *E. coli* and nitrosamine precursors group (group IV) at 9 months showing hyperplasia and dysplasia (s) in mucosal lining epithelium.

Bladder histopathological changes are presented in Figure 1. The histopathological examination revealed that *E. coli* infected group was able alone to produce some histopathological changes in bladder tissue ranging from inflammation to dysplasia and that nitrosamine precursor plus *E. coli* group showed highest incidences of urinary bladder lesions than the nitrosamine precursor group and *E. coli* group.

### NF-ÎºB p65, Bcl-2 and IL-6 levels

Levels of NF-κB p65, Bcl-2 and IL-6 are presented in Table 1. As indicated in Table 1, at 3 months interval, the mean ± SD of NF-κB p65, Bcl-2 and IL-6 levels were significantly higher in the three groups compared with those obtained in the control group (P< 0.05). In addition a significant difference was observed among the three groups, with those of group IV (receiving nitrosamine precursor and infected with *E. coli*) showing the highest values. Regarding NF-κB p65 levels, there was a significant increase in group II (0.92 ± 0.22 ng/ml), group III (0.84 ± 0.17 ng/ml), and group IV (1.19 ± 0.19 ng/ml) compared with the control group (0.57 ± 0.07 ng/ml). The anti-apoptotic protein; Bcl-2 level was significantly increased in groups II (354.74 ± 23.44 U/ml), group III (331.78 ± 11.86 U/ml), and group IV (387.05 ± 8.40 U/ml) compared with the control group level (309. 14 ± 14.55 U/ml). Finally for IL-6 level there was a significant increase in group II (19.76 ± 1.64 pg/ml), group III (19.21 ± 1.56 pg/ml), and group IV (24.80 ± 2.20 pg/ml) compared with the control group (14.09 ± 0.87 pg/ml).

**Table 1:** NF-κBp65 tissue homogenate level (ng/ml), serum level of Bcl-2 (U/ml) and IL-6 (pg/ml)

**Group**		**NF-κBp65 (ng/ml)**			**Bcl-2 (U/ml)**			**IL-6 (pg/ml)**		
		**3 months**	**6 months**	**9 months**	**3 months**	**6 months**	**9 months**	**3 months**	**6 months**	**9 months**
Group I	Range	0.51- 0.67	0.51-0.75	0.60-0.87	293.30- 331.94	310.74-331.94	305.78- 348.24	12.65-15.20	13.55-16.95	14.5-17.00
	Mean ± S.D	0.57 ± 0.07	0.61 ± 0.08	0.70 ± 0.11	309.14 ± 14.55	320.61 ± 7.47	323.47 ± 14.33	14.09 ± 0.87	14.74 ± 1.23	15.63 ± 0.89
Group II	Range	0.52-1.13	0.91- 1.145	1.13-1.74	329.75-396.42	412.63-584.94	354.27- 681.57	17.77-22.44	20.91-25.11	24.76-29.69
	Mean ± S.D	0.92 ± 0.22^a,d^	1.11 ± 0.19^a,d^	1.30 ± 0.22^a,c,d^	354.74 ± 23.44^a,d^	485.36 ± 60.12^a,c,d^	485.58 ± 120.36^a^	19.76 ± 1.64^a,d^	23.13 ± 1.46 ^a,d^	26.41 ± 1.89^a,d^
Group III	Range	0.57- 1.05	1.02-1.50	1.39- 2.09	315.83-351.77	341.94-374.09	362.57-407.20	17.22-21.78	20.08-24.22	22.50-25.59
	Mean ± S.D	0.84 ± 0.17^a,d^	1.27 ± 0.20^a,d^	1.66 ± 0.27^a,b^	331.78 ± 11.86 ^a,d^	361.59 ± 11.95^a,b,d^	386.92 ± 19.14^a,d^	19.21 ± 1.56^a,d^	22.29 ± 1.48 ^a,d^	24.37 ± 1.32^a,d^
Group IV	Range	0.94- 1.40	1.30-1.87	1.58-1.95	377.06-399.89	484.65-595.49	522.07- 726.57	22.00-27.46	26.61-34.78	35.87-43.51
	Mean ± S.D	1.19 ± 0.19^a,b,c^	1.52 ± 0.21^a,b,c^	1.72 ± 0.14^a,b^	387.05 ± 8.40^a,b,c^	544.54 ± 37.11^a,b,c^	592.60 ± 75.22^a,c^	24.80 ± 2.20^a,b,c^	30.66 ± 3.20^a,b,c^	40.55 ± 2.69^a,b,c^

Group I: Control group; Group II: *E. coli *group; Group III: Nitrosamine precursor group; Group IV: *E. coli *and nitrosamine precursor; a: Significantly different from control group (group I) at P<0.05; b: Significantly different from *E. coli *group (group II) at P<0.05; c: Significantly different from nitrosamine precursors group (group III) at P<0.05; d: Significantly different from *E. coli * + nitrosamine precursors group (group IV) at P<0.05.

At 6 months interval, the mean ± SD of NF-κB p65, Bcl-2 and IL-6 levels also showed significant increase in the three groups compared with those obtained in the control group. In addition a significant difference was also observed among the three groups, with those of group IV (receiving nitrosamine precursor and infected with *E. coli*) showing the highest values. Regarding NF-κB p65 levels, there was a significant increase in group II (1.11 ± 0.19 ng/ml), group III (1.27 ± 0.20 ng/ml), and group IV (1.52 ± 0.21 ng/ml) compared with the control group (0.61 ± 0.08 ng/ml). The anti-apoptotic protein; Bcl-2 level was significantly increased in groups II (485.36 ± 60.12 U/ml), group III (361.59 ± 11.95 U/ml), and group IV (544.54 ± 37.11 U/ml) compared with the control group level (320.61 ± 7.47 U/ml). Finally for IL-6 level there was a significant increase in group II (23.13 ± 1.46 pg/ml), group III (22.29 ± 1.48 pg/ml), and group IV (30.66 ± 3.20 pg/ml) compared with the control group (14.74 ± 1.23 pg/ml).

At 9 months interval, the mean ± SD of NF-κB p65, Bcl-2 and IL-6 levels were significantly higher in the three groups compared with those obtained in the control group. In addition a significant difference was observed among the three groups, with those of group IV (receiving nitrosamine precursor and infected with *E. coli*) showing the highest values. Regarding NF-κB p65 levels, there was a significant increase in group II (1.30 ± 0.22 ng/ml), group III (1.66 ± 0.27 ng/ml), and group IV (1.72 ± 0.14 ng/ml) compared with the control group (0.70 ± 0.11 ng/ml). The anti-apoptotic protein; Bcl-2 level was significantly increased in groups II (485.58 ± 120.36 U/ml), group III (386.92 ± 19.14 U/ml), and group IV (592.60 ± 75.22 U/ml) compared with the control group level (323.47 ± 14.33 U/ml). Finally for IL-6 level there was a significant increase in group II (26.41 ± 1.89 pg/ml), group III (24.37 ± 1.32 pg/ml), and group IV (40.55 ± 2.69 pg/ml) compared with the control group (15.63 ± 0.89 pg/ml).

## Discussion

The involvement of bacteria in the process of carcinogenesis remains controversial [6] because of a lack of agreement on potential molecular mechanisms.

It was proven that urinary tract infection promotes carcinogenesis in the urinary tract of the rat and that infection with live *E. coli* resulted in persistent infection and diffuses urothelial hyperplasia in addition to acute and chronic inflammation [12].

In our study; Group IV (nitrosamine precursor plus *E. coli* group) showed highest incidences of urinary bladder lesions than the nitrosamine precursor group; moreover *E. coli* group alone was able to produce some histopathological changes in bladder tissue. These findings suggested that urinary bladder infection by *E. coli* may play a major additive and synergistic role in bladder carcinogenesis.

These results are consistent with the study of Ashmawey and colleagues [13] who reported that *E. coli* infection in the bladder tissues increases the carcinogenic ability of nitrosamine precursors. Three mechanisms were suggested to account for the tumor enhancing effect of *E. coli* in the experiment. First, *E. coli* infection of bladder tissues increases the carcinogenic ability of nitrosamine precursors and this may be due to increase of nitrite production by the bacteria and continuous production of nitrosoamine by helping in-situ nitrosamine synthesis. Second, *E. coli* infection accelerated urothelial proliferation. This may have augmented the mutagenic effect of the carcinogen. Third, prolonged oxidative and nitrosative stresses which results in DNA damage and mutation [13].

Our results showed the highest level of NF-κBp65 in *E. coli* plus nitrosamine precursor group. In the absence of inflammatory stimuli, NF-κB p65 is retained in an inactive complex in the cytoplasm by the chaperone-like protein inhibitor of Kappa B alpha (I-κBα). With exposure to pro-inflammatory stimuli, such as Toll-like receptors-4 agonist (TLR4) or pro-inflammatory cytokines, phosphorylation of I-κB occurs, leading to its degradation and subsequent release of NF-κB p65 to translocate to the nucleus, driving inflammatory gene expression [14]. The highest level of NF-κBp65 in *E. coli* plus nitrosamine precursor group, observed in this study is in agreement with [15,16] who reported that *E. coli* lipopolysaccharide (LPS), a major cell wall component of *E. coli*, treatment induced IκB phosphorylation, IκB degradation, and NF-κB translocation. Also Saban and colleagues [17] during a study of gene expression changes occurring in the early stages of genitourinary inflammation mediated by LPS reported that NF-κB pathway genes were up-regulated by LPS stimulation. This can be explained by the fact that induction of inflammation by LPS or *E. coli* in bladder uroepithelial cells involves the TLRs and CD14. These activate signaling pathways, including NF-κB and p38 mitogen- activated protein kinase (p38 MAPKs) [18].

Our results showed highest level of Bcl-2 in *E. coli* plus nitrosamine precursor group followed by *E. coli* group and nitrosamine precursor group respectively. These results can be explained by the ability of bacterial pathogens to inhibit apoptosis in eukaryotic cells during infection as prevention of apoptosis provides a survival advantage because it enables the bacteria to replicate inside host cells [19].

Many pathogens rely on NF-κB activation to inhibit apoptosis. The Gram-negative bacteria cell surface component LPS activates the NF-κB pathway during infection [20]. Because NF-κB activation has many pro-survival effects on the host cell, activation of the NF-κB pathway by LPS might be a simple explanation of how most bacteria inhibit apoptosis [19].

The increase in Bcl-2 level in nitrosamine precursor group observed in our results is in consistent with El Gendy and colleagues [21] who reported that the levels of Bcl-2 protein significantly increased over all the periods of treatment (12 months) in rats receiving nitrosamine precursors compared with the corresponding level of normal control rats fed with standard diet.

Our results showed highest level of IL-6 in *E. coli* plus nitrosamine precursor group followed by *E. coli* group and nitrosamine precursor group respectively. *E. coli* enhancing effect on IL-6 production clearly observed in our study is in agreement with Feng and colleagues [15] who reported that LPS treatment caused a marked increase in IL-6 production in macrophages.

Neuhaus and colleagues [22] had also shown that IL-6 and IL-6 receptor expression was found in urothelium, lamina propria and detrusor cells isolated from bladder biopsies of tumor patients; these researchers further found that LPS stimulation evoked a time-dependent synthesis and/or release of IL-6, IL-6 receptor, and transcription factor signal transducer and activator of transcription 3 (Stat3) in cultured human detrusor smooth muscle cells. The ability of *E. coli* to increase IL-6 level can be explained by activating several signaling pathways, including NF-κB and p38MAPKs [18] with subsequent production of Il-6.

## Conclusion

In conclusion *E. coli* infection might play a role in the development of bladder cancer and this effect may be mediated by activation of NF-κB pathway resulting in inhibition of apoptosis and increased inflammation.

## Methods

### Experimental animals and dosing

Ninety five male albino rats, weighing 50–60 gm were included in the study and divided into four groups; as follows: group I (**Gr I, 20 rats**): Normal control fed the standard diet . Group II (**Gr II, 25 rats**): *E. coli* infected; the rats infected by 0.1 ml saline containing suspension of *E. coli* in the bladder (approximately 2 X 10 ^6^ organisms), according to [23]. Group III (**Gr III, 25 rats**): Received nitrosamine precursor; dibutyl amine (DBA) 1000 ppm and sodium nitrate 2000 ppm; in drinking water according to [21]. Group IV (**Gr IV, 25 rats**): Received nitrosamine precursor; dibutyl amine (DBA) 1000 ppm and sodium nitrate 2000 ppm; in drinking water and infected by *E. coli* in the bladder. At the end of the experiment, animals were decapitated and 5 ml of blood was collected. The present experiment was continued 36 weeks.

### Laboratory procedures

At three months interval (3, 6 and 9 months) animals were sacrificed and bladder tissue was separated and blood was collected into vacutainer clotted tubes. For histopathological studies bladder tissue pieces were fixed in 10% formalin, blocked in paraffin, sectioned, and stained with hematoxyline and eosin. Finally, the samples were examined by a pathologist.

Specimens of bladder were removed immediately from sacrificed animals, washed with saline, dried, cut into weighed pieces and kept frozen at −80°C, then tissue homogenate was prepared according to [24] for NF-κB p65 determination by ELISA kit (Glory Science Co., Ltd, USA) following the manufacturer instructions.

Sera were obtained by centrifugation at 4000 rpm for 10 minutes. Sera were separated. Aliquoted sera were kept frozen at −80°C until used for Bcl-2 determination by ELISA kit (the Calbiochem Laboratories, USA, Cat QIA23) and IL-6 determination by ELISA kit (IBL, USA, Cat IB39452) following the manufacturer instructions.

### Statistical analysis

All statistical analyses were performed using GraphPad Prism version 5.01 software package (GraphPad Software, Inc. CA, USA). Data are presented as mean ± standard deviation (S.D). To determine differences between groups, analysis of variance (ANOVA) followed by Tukey’s multiple comparison post hoc analysis was used for multiple comparisons between different groups. The level of statistical significance was set at probability P ≤ 0.05.

## Abbreviations

*E. coli*: Escherichia coli; NF-κBp65: Nuclear factor kappa p65; Bcl-2: B-cell lymphoma 2; IL-6: Interleukin 6; UTIs: Urinary tract infections; IL-1β: Interleukin 1β; TNF: Tumor necrosis factor; EGF: Epidermal growth factor; I-κBα: Inhibitor of Kappa B alpha; TLR4: Toll-like receptors-4; LPS: Lipopolysaccharide; CD14: Cluster of differentiation 14; p38 MAPKs: p38 mitogen- activated protein kinase; Stat3: Signal transduction and transcription 3; DBA: Dibutyl amine; S.D: Standard deviation; ANOVA: Analysis of variance.

## Competing interests

The authors declare that they have no competing interests.

## Authors’ contributions

HE: Wrote the manuscript and participated in the study design. TS: Revised the manuscript and assisted with data analysis. AS: participated in the study design and supervised the ELISA work. NO: Performed the ELISA work and performed the data analysis. All authors read and approved the final manuscript.
